# Changes of Local Blood Flow in Response to Acupuncture Stimulation: A Systematic Review

**DOI:** 10.1155/2016/9874207

**Published:** 2016-06-14

**Authors:** Song-Yi Kim, Seorim Min, Hyangsook Lee, Soyeon Cheon, Xiuyu Zhang, Ji-Yeun Park, Taek-Jin Song, Hi-Joon Park

**Affiliations:** ^1^Studies of Translational Acupuncture Research, Acupuncture and Meridian Science Research Center, Kyung Hee University, 26 Kyungheedae-ro, Dongdaemun-gu, Seoul 02447, Republic of Korea; ^2^Department of Korean Medical Science, Graduate School of Korean Medicine, Kyung Hee University, Seoul, Republic of Korea; ^3^College of Korean Medicine, Daejeon University, 62 Daehak-ro, Dong-gu, Daejeon 34520, Republic of Korea

## Abstract

*Objectives*. This systematic review aimed to summarize and evaluate the findings of studies investigating the local microcirculatory effects following acupuncture stimulation.* Methods*. MEDLINE, EMBASE, OASIS, and Cochrane library were searched to identify randomized controlled trials (RCTs) published before January 30, 2015. Studies demonstrating any type of microcirculation response to manual acupuncture in healthy subjects and patients were included. The risk of bias and the reliability of the experimental conditions were evaluated to determine quality assessment.* Results*. Eight RCTs met the inclusion criteria; there was at least one acupuncture-induced change in a microcirculatory parameter. Of the seven studies in healthy subjects, four reported significant increases in blood flow following acupuncture compared with control, whereas one other study observed reductions in microcirculation immediately after acupuncture needling. The studies that assessed patients with either fibromyalgia or trapezius myalgia found significant increases in blood flow in the skin and muscle. Additionally, the degree and duration of increases in microcirculation varied depending on the condition of the subjects and the manipulation technique.* Conclusions*. The current evidence regarding the local effects of acupuncture in terms of blood flow remains insufficient for reliable conclusions due to few well-designed studies. Additional well-designed studies are needed to clarify these issues.

## 1. Introduction

Acupuncture is a practical and cost-effective intervention with few adverse side effects that is useful for the treatment of many disorders. In particular, acupuncture treatment has been shown to have analgesic effects in patients with various pain conditions, such as low back pain, headaches, and osteoarthritis [1]. Although the detailed mechanisms underlying acupuncture remain poorly understood, several lines of evidence suggest that local molecular and cellular changes occur at and around the location of the needled acupoint. For example, the activities of adenosine, which is a neuromodulator with antinociceptive properties [2], and the extracellular signal-regulated kinase (ERK) pathway [3], which is involved in the local mechanisms associated with acupuncture, are altered following treatment and might mediate the varied effects of acupuncture, including analgesia. Additionally, it has also been suggested that the morphological changes induced by acupuncture in connective tissue and fibroblasts are involved in this process [4, 5].

Microcirculation is affected by the autonomic nervous system (ANS) and is regarded as a useful method with which to evaluate the peripheral effects of acupuncture [6–8]. In an animal model [9], the treatment resulted in changes in group II, III, and IV afferent nerve fibers and/or the efferent nerve pathway, which includes intrinsic cholinergic vasodilators originating from the basal forebrain, pelvic parasympathetic cholinergic vasodilator nerves, and the calcitonin gene-related peptide (CGRP) system. Moreover, these changes were associated with alterations in vertebral, uterine, and skeletal muscle blood flow. The cutaneous microcirculation system is complex and dynamic [10], and different responses of blood flow in the skin or muscle, especially at painful locations, can be elicited depending on the particular condition of the subject [11, 12]. However, the current evidence detailing the relationship between changes in blood flow and therapeutic effects remains insufficient, and the physiological understanding of blood perfusion following acupuncture treatment remains incomplete. On the other hand, studies measuring these types of physiological changes have recently increased due to efforts to determine the effects of acupuncture.

Thus, the primary aims of the present review were to summarize and evaluate the effects of acupuncture on local microcirculation and to compare the blood flow changes induced by acupuncture among five types of typical control groups: no-acupuncture, nonpenetrating acupuncture at same acupoint, superficial acupuncture, acupuncture needling at a nonacupoint, and insertion-only without manipulation.

## 2. Methods

### 2.1. Study Selection

The present review conducted a literature search of the Cochrane library, MEDLINE, EMBASE, and Oriental Medicine Advanced Searching Integrated System (OASIS) databases for studies investigating acupuncture-induced changes in local blood flow that were published up to January 30, 2015. The search was restricted to human studies using the following terms in combination: “acupuncture, ear acupuncture, moxibustion, meridian or acupoint^*∗*^” and “blood flow, microcirculation, Doppler, near infrared spectroscopy, near infrared, or perfusion.” Additionally, the reference lists of the selected studies and relevant reviews were also manually searched to identify other appropriate studies.

The present review only included original randomized controlled trials (RCTs) that were written in English, Chinese, or German. The details of the inclusion criteria are as follows: (1) the study participants were healthy subjects or patients, (2) the study employed manual needle acupuncture at acupoint(s) and compared the results with a sham control group (nonpenetrating acupuncture, superficial acupuncture, or nonacupoint insertion), insertion-only without manipulation or a subject who did not receive acupuncture, and (3) the outcome included any microcirculatory response to acupuncture including volume, velocity, blood flow, blood flow resistance, and blood vessel diameter around the stimulus sites. Studies were excluded from the present analysis if (1) the intervention included acupuncture-related techniques without skin penetration or did not employ manual acupuncture (electroacupuncture, laser acupuncture, transcutaneous electrical nerve stimulation, acupressure, or moxibustion) and/or (2) the outcome measures did not focus on changes in local blood flow.

### 2.2. Data Extraction

The data extraction was performed by four reviewers (Song-Yi Kim, Seorim Min, Soyeon Cheon, and Xiuyu Zhang) using a predefined form. Research characteristics such as study design, population, intervention and control groups, outcome measures, and comparisons of the blood flow results between study groups were extracted from each article. Two other reviewers (Hi-Joon Park and Hyangsook Lee) independently verified the extracted data.

### 2.3. Quality Assessment

To assess the methodological quality of the selected studies, two types of checklists were used. First, a quality assessment was performed by two reviewers (Song-Yi Kim and Ji-Yeun Park) using a modified version of the tool for the assessment of risk of bias (RoB) from the Cochrane Handbook [21]. Selection, performance, detection, attrition, and reporting biases were estimated using the six items of modified assessment tool; an answer of “yes (Y),” “?,” and “no (N)” indicated a low RoB, a lack of information with which to judge the RoB, and a high RoB, respectively. Second, to assess the reliability of the experimental results, the confounding variables and details of the experimental environment that could influence microcirculation were assessed with a checklist developed by the present authors. This checklist was composed of items evaluating study design (for a cross-over design, washout time was considered), characteristics of the study participants (age, gender, and acupuncture experiences), measurement accuracy (posture during measurement, movement control, and details concerning rest before measurement and restrictions prior to the experiment), and the characteristics of the experiment environment (temperature, humidity, light, and other types of control). Two reviewers (Seorim Min and Taek-Jin Song) conducted the assessment using this checklist. The above two assessments of methodological quality were independently cross-checked by two reviewers (Song-Yi Kim and Seorim Min), and disagreements were resolved by a discussion involving all the authors.

## 3. Results

### 3.1. Results of Search

**Figure 1:**
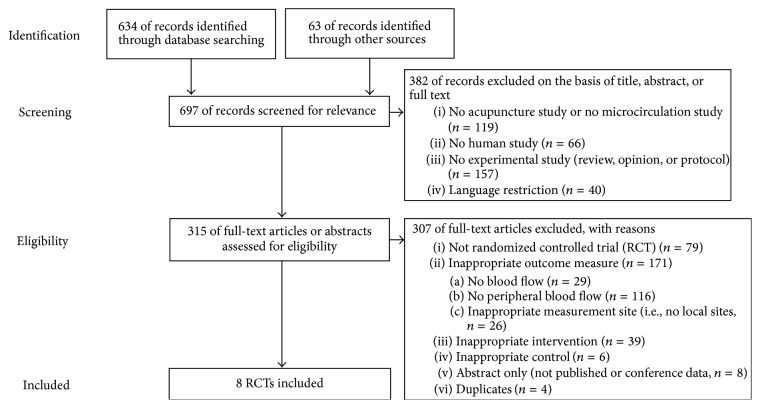
Flowchart.

The electronic database searches and manual selection of additional experiments identified 697 studies that fit the inclusion criteria. After the exclusion of non-English/Chinese/German-language publications and studies that did not use human subjects based on their titles and abstracts, 315 studies remained eligible for further assessment; of these, eight were RCTs that were ultimately included in the present review (Figure 1).

### 3.2. Study Design and Participants

The eight selected RCTs included a total of 205 participants that met the inclusion criteria of the present review; the characteristics of the studies are summarized in Table 1 [13–20]. All studies used a cross-over design. All participants in the selected studies were healthy [13–17, 19, 20], with the exception of those with fibromyalgia [17, 18] and trapezius myalgia [17], who participated in two studies.

### 3.3. Acupuncture Intervention and Control Groups

In most of the selected studies, acupuncture was performed at points located distal to the elbows and knees such as LI4 [13, 15], ST36 [18, 19], and PC6 [14, 20], whereas one study simultaneously used several acupoints on the extremities [16]. In one study [17], the GB21 acupoint on the posterior region of the shoulder was stimulated with an acupuncture needle. Five types of control group were included in the present review. There were eight sham-controlled studies (11 comparisons) including two with nonpenetrating acupuncture at the same acupoints [14, 16], four with superficial needling at the same acupoints [14, 17–19], three with identical needling at nonacupoints [14, 15, 20], and two with insertion-only without manipulation at the same acupoint [13, 19]. Additionally, no-acupuncture groups were used as a control in three of the studies [17–19].

### 3.4. Primary Outcome Measures

#### 3.4.1. Measurement Methods

The primary outcome measure was changes in skin or muscle blood flow compared with the control group. To measure the local effects of acupuncture, the measurement sites were stimulated at an acupoint, a region adjacent to the acupoint, or the nearby fingertip. The selected studies assessed outcomes relevant to blood flow, including the blood perfusion ratio, microcirculatory volume, velocity of blood flow, and capillary caliber. The studies selected for the present review used various measurement instruments including laser Doppler perfusion imaging (LDPI) with a PIM system (Perimed, Sweden; wavelength, sampling depth: unknown [15], wavelength: 670 nm, sampling depth: 0.5–1 mm [14, 20], wavelength: 658 nm, sampling depth: unknown [13]), laser Doppler flowmetry (LDF) with a FLO-N1 (Omegawave, Japan; wavelength: 780 nm, sampling depth: about 1 mm [16]), and two types of custom photoplethysmographic (PPG) techniques (wavelength: 560 nm, sampling depth: 1-2 mm [17–19]). To measure changes in muscle blood flow, two types of custom-designed PPG techniques were used (wavelength: 804 nm, sampling depth: 13.6 mm [17] and wavelength: 880 nm, sampling depth: 13.0 mm [17–19]).

#### 3.4.2. Microcirculatory Changes

All eight studies selected for the present review observed acupuncture-induced changes in blood flow in the skin (Table 2), whereas three studies observed changes in blood flow in the muscle (Table 3). Of the seven studies that assessed healthy subjects, four reported significant increases in blood flow compared with the no-acupuncture [17, 19], nonpenetrating acupuncture [16], superficial acupuncture [17, 19], and insertion-only control [13, 19] groups. Compared with the nonacupoint control groups, acupuncture treatment did not induce statistically significant differences in blood flow in two studies [14, 15], whereas reductions in blood flow immediately after acupuncture needling were observed in one other study [20]. In the studies that assessed patients with fibromyalgia [17, 18] and trapezius myalgia [17], the sensitivity of microcirculation to acupuncture needling varied according to patient characteristics.

Sandberg's group published three consecutive papers [17–19] comparing blood flow changes in the skin and muscles of healthy subjects with those of patients with either fibromyalgia or trapezius myalgia after acupuncture stimulation at ST36 or GB21. Deep acupuncture at ST36 on the tibial muscle induced greater change in skin and muscle blood flow than superficial acupuncture (subcutaneously inserted needle) in both healthy subjects and fibromyalgia patients. However, the degree of increased blood flow induced by superficial acupuncture was much higher in the fibromyalgia patients than in the healthy subjects [18]. The subsequent study from this group [17] revealed that acupuncture performed at GB21 (on the back of the shoulder located on the trapezius muscle), where the trapezius myalgia and fibromyalgia patients suffered pain, produced different patterns of microcirculation. As in their previous study using ST36 [19], deep acupuncture in healthy subjects induced greater changes in skin and muscle blood flow than superficial acupuncture. However, superficial acupuncture was as effective as or more effective than deep muscle stimulation in inducing an increase in blood flow in the skin and muscles of the fibromyalgia patients whereas the reverse was the case in healthy subjects [18, 19]. Furthermore, the increased blood flow in the muscle induced by superficial acupuncture in fibromyalgia patients lasted for 60 minutes, which was longer than the duration in healthy subjects (40 minutes). In contrast, the trapezius myalgia patients exhibited a different pattern of altered blood flow compared with the healthy subjects and fibromyalgia patients; specifically, trapezius myalgia patients reacted to both deep and superficial acupuncture with similar changes in skin and muscle blood flow and smaller degrees of change and the duration of their changes was shorter [17].

Litscher et al. [20] reported a transient reduction in skin microcirculation following acupuncture at a nearby fingertip and the transient effects of acupuncture stimulation on transient sympathetic activation.

One study [15] observed local perfusion on the dorsum of a hand after stimulation at LI4 or at a nonacupoint. They showed that the trend toward an increase in perfusion was similar regardless of whether the stimulation was at an acupoint or a nonacupoint. However, it was found that acupoint stimulation induced increases in perfusion around the adjacent areas along the Meridian system (Large Intestine Meridian), whereas nonacupoint stimulation did not.

### 3.5. Other Related Findings

Tsuchiya et al. [16] found that the plasma concentrations of nitric oxide (NO) obtained from the acupunctured arm of subjects exhibited a significant increase at 5 minutes and 60 minutes after acupuncture and that there was a correlation between increased palmer blood flow and NO concentration. Min et al. [13] investigated the dose-dependent effects of acupuncture on skin perfusion and found that repeated acupuncture manipulations (×3 manipulations) enhanced microcirculatory perfusion to a greater degree than single-manipulation (×1 manipulation) and insertion-only (0 manipulations) acupuncture. These authors also found that repeated acupuncture manipulations significantly reduced pressure pain at ST25 and reported a significant negative correlation between changes in local perfusion and pressure pain.

### 3.6. Methodological Quality

#### 3.6.1. Risk of Bias

The findings of the present methodological quality assessment are summarized in Table 4. One RCT [13] used a random table in Excel, one RCT [14] used the drawing of lots to allocate patients to experimental groups, and all six RCTs failed to report information regarding allocation concealment. In terms of participant and practitioner blinding, all studies exhibited a high or unclear RoB, except for two [14, 16] that utilized a participant blinding procedure. Three studies [17–19] recorded the needling pain sensation of each group, but it was not possible to judge whether each of these studies had a low or high RoB due to insufficient information. Because the measurement of blood perfusion is generally considered an objective outcome, outcome-assessor blinding was regarded to be a low risk in all included studies. Additionally, all studies reported incomplete outcome data [13–20]; in terms of selective reporting, one study [14] received a rating of “No” because they omitted a key outcome that would have been expected to be reported as part of a group comparison.

#### 3.6.2. Experiment Accuracy Assessment

Table 5 and Supplementary Data 1 provide the details of the study characteristics and the experimental environments in terms of the assessment of the accuracy of each experiment. All the cross-over studies except one [14] reported washout periods between the acupuncture treatments. Similarly, the age and gender of the participants were well described in all eight studies, but four [13–15, 20] did not report the acupuncture experience of the participants. Only one study satisfied all the checklist items regarding measurement accuracy [13]; in particular, details regarding the participants' movement control were poorly reported. Prior to the experiments, the participants in the selected studies were primarily asked to restrict medications, smoking, caffeine, food, and/or exercise, but these restrictions were in force for various periods of time. It was assumed that a constant room temperature was maintained, but most studies did not clearly report humidity and light levels during experiments.

## 4. Discussion

The results of the present review revealed that the eight eligible studies reported a change in at least one microcirculatory parameter following acupuncture treatment. Of the seven studies that assessed healthy subjects, four reported significant increases in blood flow compared with the no-acupuncture, nonpenetrating acupuncture, superficial acupuncture, or insertion-only control groups [13, 16, 17, 19]. In two studies [14, 15], acupuncture did not induce statistically significant changes compared with the nonacupoint control groups; however, one of these two studies [15] reported an increase in skin perfusion in the adjacent area rather than in the local area. One study [20] observed a reduction in blood flow immediately after acupuncture needling, which is indicative of transient sympathetic activation. In the two studies of patients with fibromyalgia or trapezius myalgia [17, 18], there were significant increases in blood flow in the skin and muscle, but the sensitivity of microcirculation to the acupuncture needling varied depending on the characteristics of disease. Additionally, the degree and duration of the local increases in microcirculation varied depending on the condition of the subjects (healthy subjects versus patients) and the manipulation technique (repeated manipulation and depth of needling).

### 4.1. Clinical Aspects of Microcirculation Measures: Differences between Healthy Subjects and Patients in Microcirculation Changes after Acupuncture

Seven of the eight selected studies reported increases in at least one microcirculatory parameter after acupuncture treatment and, of these changes, the most interesting findings tended to involve discrepancies between healthy subjects and patients [17, 18]. According to Sandberg et al. [17], deep acupuncture stimulation to the painful muscle in trapezius myalgia and fibromyalgia patients caused a smaller increase in muscle blood flow than it did in healthy subjects who had no pain at the acupuncture sites. Several studies have demonstrated that trapezius myalgia patients with chronic neck pain have low local blood flow in the painful side [11], and it has been suggested that the impaired regulation of cutaneous microcirculation might be one of the predisposing factors underlying inflammation and ulceration in chronic venous insufficiency [22]. Mitochondrial disturbances and/or metabolic abnormalities in the trapezius muscle might also explain the low microcirculatory response in patients with work-related trapezius myalgia or fibromyalgia [11, 17, 23].

Invasive interventions, such as low-frequency transcutaneous electrical nerve stimulation (TENS) [24, 25] and glutamate injections into either latent myofascial trigger points (MTP) or non-MTP points [26], also affect local cutaneous blood flow. Interestingly, nociceptive stimulation onto latent MTPs in healthy subjects induced significantly lower increases in blood flow in the local area compared with that onto non-MTPs due to the activation of vasoconstriction mechanisms [26]. These results are comparable to the present findings, because MTPs represent one of the acupoints known as an Ashi point (ouch point), which is determined by pathological responses such as tenderness [27]. Overall, these findings indicate that the measurement of blood flow in the skin and muscle in response to acupuncture stimulation may be an additional surrogate outcome measure in clinical trials of pain.

Local reactions (i.e., deqi sensations) induced by acupuncture manipulations are believed to be an important factor in the induction of its effects. Min et al. [13] studied whether acupuncture-induced changes in local microcirculation could have a clinical influence and found that the repeated manipulation of LI4 enhanced local microcirculation and increased the analgesic effects compared with single manipulations or insertion-only. Additionally, these two parameters were correlated, which indicates that local increases in microcirculation might influence the analgesic effects of acupuncture.

### 4.2. Transient Reduction in Blood Flow following Acupuncture due to Initial Increases in Sympathetic Activity

Litscher et al. [20] reported a reduction in skin microcirculation immediately following acupuncture treatment. Kimura et al. [28] and Kistler et al. [29] also found that acupuncture increased sympathetic activity and transiently reduced blood flow in the skin, but these parameters subsequently returned to baseline levels. Litscher et al. [20] also reported that acupuncture induced an immediate reduction in microcirculation that returned to normal levels within 1 minute. Kang et al. [30] recently reported that both verum and sham acupuncture resulted in increases in the skin conductance response (SCR) and heart rate within tens of seconds. They also suggested that the increase in SCR in conjunction with cardiac deceleration strongly resembles the main characteristics of the so-called orienting response, which is evoked when an organism is initially confronted with a novel, threatening cue [19, 30]. On the other hand, the initial autonomic response patterns induced by acupuncture could be explained by the uncertainty of the participants and the enhanced fear of pain [30], because these kinds of immediate responses to acupuncture seem to be differentiated from the long-term physiological responses. Sandberg et al. [19] suggested that the transient reduction in blood flow following the activation of sympathetic neurons during this process may be masked by the more powerful effects of increased levels of vasodilative factors.

### 4.3. Consideration of the Placebo Effect in Acupuncture: Microcirculatory Changes following Superficial Needling

Although the establishment of proper control procedures in the field of acupuncture research is essential, the issues associated with acupuncture control remain difficult and the focus of contentious debate [6, 31]. Various strategies can be used to select control groups. For example, control stimulation via the use of superficial needling, in which the skin is penetrated without the deqi sensation, remains highly controversial with respect to whether it is actually an inert placebo [32]. Indeed, superficial needling has a comparable effect under conditions of pain, such as migraine [33]. In terms of physiological changes, several studies in the present review [17–19] found that both deep and superficial needling generated significantly greater blood flow increases compared with those found in the no-acupuncture groups. One of these studies reported that superficial acupuncture in the lower limb (ST36) resulted in greater increases in blood flow in the anterior tibial muscle and skin of fibromyalgia patients than in healthy subjects, but no differences were found when deep acupuncture was used [18]. Another study [17] performed acupuncture treatment at GB21 and found that superficial acupuncture induced greater elevations of blood flow in the trapezius muscle and skin compared with deep acupuncture in fibromyalgia and trapezius myalgia patients but not in healthy subjects.

Taken together, these findings indicate that there are a generally higher sensitivity to pain and other abnormalities during the perception of somatosensory information in patients with pain and that these result in different responses to superficial acupuncture based on the particular conditions of the subject. Additionally, these findings suggest that, in the case of selecting acupoints close to the disease site, minimal stimuli, such as superficial needling, may be superior to deep needling in terms of increasing skin and muscle blood flow in fibromyalgia patients. Thus, the results suggest that comparisons of acupuncture with superficial acupuncture should be undertaken with caution due to the contradictory physiological effects depending on the patient's condition. It is possible that superficial acupuncture could be considered as a viable intervention as different doses or separate stimulation methods under certain circumstances.

### 4.4. Suggested Mechanisms Underlying Microcirculatory Changes after Acupuncture

The studies selected for the present review propose several possible mechanisms that may underlie the changes in microcirculation following acupuncture treatment. Acupuncture-induced neural signaling is thought to result in alterations of blood perfusion via the local action of vasodilative factors such as substance P and CGRP [13, 15, 17–19]. One study indicated that NO-dependent mechanisms are involved in this process based on the correlation between increased blood flow and increased levels of NO in the acupuncture group [16]. It suggested here that the increased blood perfusion induced by acupuncture stimulation might be relevant to the suppression of the sympathetic nerve activity and the vasodilation in local microvascular beds.

### 4.5. Limitations

The present review has several limitations. First, there were heterogeneities among the selected studies in terms of populations, intervention methods (acupoints, depth, and manipulation), control groups, and outcome measures that made reliable comparisons difficult. In particular, the assessment of outcome measures widely varied in terms of measurement devices, outcomes, measurement sites, and measurement times; thus, it was not possible to pool the data. Second, during the evaluation of the accuracy of each experiment, it was not possible to include sufficient information regarding whether each study was properly performed. Thus, the present review assessed only whether items related to the precision of the experiment were reported and, therefore, the results of this review should be interpreted with caution. Finally, there were methodological shortcomings of included studies such as the use of small sample sizes and a lack of information concerning sequence generation and allocation concealment, and these factors need to be seriously considered to design further studies.

## 5. Conclusions

The present review determined that acupuncture induces transient reductions in blood flow due to sympathetic activation that are followed by subsequent increases in local blood flow in the skin and/or muscle. Additionally, the depth of needling and the condition of the patient may result in different microcirculatory changes, which suggests that acupuncture may be used as an additional surrogate outcome measurement in pain studies and underscores the remaining questions about whether placebo acupuncture is actually physiologically inert. Other factors to consider include the dose of acupuncture, because it may act as a confounding variable when comparing studies investigating changes in skin blood flow. The current evidence regarding the local effects of acupuncture on blood flow remains too limited to draw firm conclusions due to the small number of well-designed studies. Future well-designed studies are necessary to address these issues.

## Supplementary Material

Details regarding study design, participants' characteristics, measurement accuracy, and experimental environment are shown.

## Figures and Tables

**Table 1:** Summary of the included studies.

Author (year) country	Study design	Population (number participants)	Group		Outcome measurement			
			Acupuncture (depth, manipulation, deqi)	Control	Device	Outcome	Measurement site	Measurement time
Min et al. (2015) Korea [13]	RCT, cross-over	HS (12)	LI4 (15 mm depth, manipulation with deqi, three repeated manipulations)	Insertion-only: same acupoint as EXP	LDPI	Ratio of relative changes in perfusion^a^	LI4	Before, 0–7, 7–14, 14–21 min after needle insertion, 0–5 and 5–10 min after needle withdrawal

Huang et al. (2012) China [14]	RCT, cross-over	HS (29)	PC6 (manipulation with deqi)	(A) Superficial needing: same acupoint as EXP without manipulation (B) Nonacupoint: 1 cm from same acupoint as EXP, manipulation with deqi (C) Nonpenetrating control: same acupoint as EXP (empty tube)	LDPI	Ratio of relative changes in perfusion^a^	(A) Meridian (PC6–PC3) (B) PC3 (the elbow joint)	Before, immediately after, 5, 10 min after needle insertion, immediately after, 10 min after needle withdrawal

Zhang et al. (2008) China [15]	RCT, cross-over	HS (20)	LI4 (manipulation with deqi)	Nonacupoint: nearby LI4	LDPI	Relative change rates	(A) LI4 (B) Nonacupoint (near LI4) (C) Forefinger tip	Before, 0–5, 5–10, 10–15, 15–20, 20–25 min after needle insertion, 0–5 and 5–10 min after needle withdrawal

Tsuchiya et al. (2007) Japan [16]	RCT, cross-over	HS (20)	LI4, PC6, PC8, LU6, HT7 (5~8 mm depth, manipulation with deqi)	Nonpenetrating control: same acupoints as EXP (empty tube)	Laser tissue-blood flow meter	Skin blood flow (mL/min/100 g of tissue)	Center of the palm	Before, 5 and 60 min after needle withdrawal

Sandberg et al. (2005) Sweden [17]	RCT, cross-over	HS, patients (fibromyalgia, trapezius myalgia) (44)	GB21 (~10 mm depth, with deqi)	(A) Superficial needing: same acupoint as EXP without manipulation (B) No-acupuncture	PPG	Relative changes in skin and muscle blood flow (%)	Trapezius and supraspinatus muscle and skin	Before, 0–5 and 5–20 min after needle insertion, 0–20 and 20–40 min after needle withdrawal

Sandberg et al. (2004) Sweden [18]	RCT, cross-over	Patients (fibromyalgia) (15)	ST36 (~20 mm depth, manipulation with deep sensation)	(A) Superficial needing: same acupoint as EXP (~2-3 mm depth) (B) No-acupuncture	PPG	Relative changes in skin and muscle blood flow (%)	Around the ST36 (tibialis anterior muscle and skin)	Before, 0–5 and 5–20 min after needle insertion

Sandberg et al. (2003) Sweden [19]	RCT, cross-over	HS (14)	ST36 (~20 mm depth, with deqi)	(A) Insertion-only: same acupoint as EXP (~20 mm depth, without deqi) (B) Superficial needing: same acupoint as EXP (~2-3 mm depth) (C) No-acupuncture	PPG	Relative changes in skin and muscle blood flow (%)	Around the ST36 (tibialis anterior muscle and skin)	Before, 0–5 and 5–20 min after needle insertion

Litscher et al. (2002) Austria [20]	RCT, cross-over	HS (51)	PC6 (5 mm depth, with deqi, no manipulation)	Nonacupoint: lateral from the radius 3 cm above the carpal fold	LDPI	Mean perfusion (volt)	Middle fingertip	Before, immediately after, 1 min after needle insertion

^a^Normalised data to remove the influence of the whole effect.

EXP, experimental group; HS, healthy subjects; LDPI, laser Doppler perfusion imaging; min, minutes; PPG, photoplethysmography; RCT, randomized controlled trials.

**Table 2:** Skin blood flow results of comparison between groups.

Author (year)	Immediately after acupuncture insertion or manipulation		During acupuncture	After acupuncture withdrawal	Follow-up
	(within 1 min)	(within 5 min)			
Acupuncture versus no-acupuncture					
Sandberg et al. (2005) [17]		HS: *p* < 0.001 (↑) FM: *p* < 0.001 (↑) TM: *p* = 0.030	HS: *p* < 0.001 (↑) FM: *p* = 0.003 (↑) TM: NS	HS: *p* = 0.001 (↑) FM: NS TM: NS	HS: NS FM: NS TM: NS
Sandberg et al. (2004) [18]		FM: *p* = 0.001 (↑)	FM: *p* = 0.002 (↑)	—	—
Sandberg et al. (2003) [19]		*p* = 0.003 (↑)	NS	—	—

Acupuncture versus nonpenetrating acupuncture (same acupoint)					
Huang et al. (2012) [14]		NS	NS	NS	NS
Tsuchiya et al. (2007) [16]	—	—	—	*p* < 0.05 (↑)	*p* < 0.05 (↑)

Acupuncture versus superficial acupuncture					
Huang et al. (2012) [14]		NS	NS	NS	NS
Sandberg et al. (2005) [17]	—	HS: NS FM: *p* < 0.001 (↑) (Sup > Deep)^*∗*^ TM: NS	HS: *p* < 0.001 (↑) FM: NS TM: NS	HS: *p* = 0.001 (↑) FM: NS TM: NS	HS: NS FM: NS TM: NS
Sandberg et al. (2004) [18]	—	FM: *p* = 0.001 (↑)	FM: *p* = 0.002 (↑)	—	—
Sandberg et al. (2003) [19]	—	*p* = 0.003 (↑)	NS	—	—

Acupoint versus nonacupoint					
Huang et al. (2012) [14]	—	NS	NS	NS	NS
Zhang et al. (2008) [15]	—	NS	NS	NS	—
Litscher et al. (2002) [20]	*p *< 0.001 (↓)	—	NS	—	—

Acupuncture with manipulation versus insertion-only					
Min et al. (2015) [13]	—	—	*p* < 0.01 (↑)	NS	—
Sandberg et al. (2003) [19]	—	*p *= 0.003 (↑)	NS	—	—

^*∗*^Superficial acupuncture had greater increase of skin blood flow than deep acupuncture; FM, fibromyalgia patients; HS, healthy subjects; NS, not significantly different; TM, trapezius myalgia patients. Most statistically significant values indicate the increase of blood flow compared to the control. “↑” means increase of blood flow compared to the control. “↓” means decrease of blood flow compared to the control. “—” means “not reported” or “not applicable.”

**Table 3:** Muscle blood flow results of comparison between groups.

Author (year)	Immediately after acupuncture insertion or manipulation	During acupuncture	After acupuncture withdrawal	Follow-up
Acupuncture versus no-acupuncture				
Sandberg et al. (2005) [17]	HS: *p* < 0.001 (↑) FM: *p* < 0.001 (↑) TM: *p* = 0.009 (↑)	HS: *p* < 0.001 (↑) FM: *p* = 0.001 (↑) TM: NS	HS: *p* = 0.002 (↑) FM: *p* = 0.002 (↑) TM: NS	HS: NS FM: NS TM: NS
Sandberg et al. (2004) [18]	FM: *p* < 0.001 (↑)	FM: *p* < 0.001 (↑)	—	—
Sandberg et al. (2003) [19]	*p* = 0.001 (↑)	*p* = 0.007 (↑)	—	—

Acupuncture versus superficial acupuncture				
Sandberg et al. (2005) [17]	HS: NS FM: *p* < 0.001 (↑) (Sup > Deep)^*∗*^ TM: NS	HS: *p* < 0.001 (↑) FM: NS TM: NS	HS: *p* = 0.002 (↑) FM: NS TM: NS	HS: NS FM: NS TM: NS
Sandberg et al. (2004) [18]	FM: *p* < 0.001 (↑)	FM: *p* < 0.001 (↑)	—	—
Sandberg et al. (2003) [19]	*p* = 0.001 (↑)	*p* = 0.007 (↑)	—	—

Acupuncture with manipulation versus insertion-only				
Sandberg et al. (2003) [19]	*p* = 0.001 (↑)	*p* = 0.007 (↑)	—	—

^*∗*^Superficial acupuncture had greater increase of blood flow than deep acupuncture; FM, fibromyalgia patients; HS, healthy subjects; NS, not significantly different; TM, trapezius myalgia patients. “↑” means increase of blood flow compared to the control. “—” means “not reported” or “not applicable.”

**Table 4:** Methodological quality of the included studies.

Author (year)	Selection		Performance	Detection	Attrition	Reporting
	1	2	3	4	5	6
Min et al. (2015) [13]	Y	?	?	Y	Y	Y
Huang et al. (2012) [14]	Y	?	Y	Y	Y	N
Zhang et al. (2008) [15]	?	?	?	Y	Y	Y
Tsuchiya et al. (2007) [16]	?	?	Y	Y	Y	Y
Sandberg et al. (2005) [17]	?	?	?/N^a^	Y	Y	Y
Sandberg et al. (2004) [18]	?	?	?/N^a^	Y	Y	Y
Sandberg et al. (2003) [19]	?/N^a^	?	?/N^a^	Y	Y	Y
Litscher et al. (2002) [20]	?	?	?	Y	Y	Y

Items for RCT: 1, adequate sequence generation; 2, adequate allocation concealment; 3, blinding of participants and research personnel; 4, blinding of outcome assessments; 5, adequate consideration of incomplete outcome data; 6, free of suggestion of selective outcome reporting [21].

N, no (high risk of bias); ?, unclear (lack of information); Y, yes (low risk of bias).

^a^Other control groups/no-acupuncture group.

**Table 5:** Checklist for study characteristics and experimental environments.

Author (year)	Design	Participants characteristics			Measurement accuracy				Experiment environment			
	1	2	3	4	5	6	7	8	9	10	11	12
Min et al. (2015) [13]	Y	Y	Y	—	Y	Y	Y	Y	Y	—	Y	Y
Huang et al. (2012) [14]	—	Y	Y	—	—	Y	Y	—	Y	Y	—	NA
Zhang et al. (2008) [15]	Y	Y	Y	—	—	—	—	—	Y	—	—	NA
Tsuchiya et al. (2007) [16]	Y	Y	—	Y	—	—	—	Y	—	—	—	NA
Sandberg et al. (2005) [17]	Y	Y	Y	Y	Y	Y	—	Y	Y	—	Y	Y
Sandberg et al. (2004) [18]	Y	Y	Y	Y	Y	—	Y	Y	Y	—	Y	Y
Sandberg et al. (2003) [19]	Y	Y	Y	Y	Y	—	Y	Y	Y	—	Y	Y
Litscher et al. (2002) [20]	Y	Y	Y	—	Y	—	Y	Y	—	—	—	NA

Items: 1, for cross-over design, washout time considered; 2, description of participants' age; 3, description of participants' gender; 4, description of participants' experience for acupuncture; 5, description of posture during measurement; 6, description of movement control; 7, description of details of rest before measurement; 8, description of restriction before experiment; 9, description of temperature control; 10, description of humidity control; 11, description of light control; 12, others. NA, not applicable; Y, item described; —, not reported.

## References

[1] Vickers A. J., Cronin A. M., Maschino A. C. (2012). Acupuncture for chronic pain: individual patient data meta-analysis. *Archives of Internal Medicine*.

[2] Goldman N., Chen M., Fujita T. (2010). Adenosine A1 receptors mediate local anti-nociceptive effects of acupuncture. *Nature Neuroscience*.

[3] Park J.-Y., Park J. J., Jeon S. (2014). From peripheral to central: the role of erk signaling pathway in acupuncture analgesia. *The Journal of Pain*.

[4] Langevin H. M., Bouffard N. A., Badger G. J., Churchill D. L., Howe A. K. (2006). Subcutaneous tissue fibroblast cytoskeletal remodeling induced by acupuncture: evidence for a mechanotransduction-based mechanism. *Journal of Cellular Physiology*.

[5] Langevin H. M., Churchill D. L., Cipolla M. J. (2001). Mechanical signaling through connective tissue: a mechanism for the therapeutic effect of acupuncture. *FASEB Journal*.

[6] Moffet H. H. (2006). How might acupuncture work? A systematic review of physiologic rationales from clinical trials. *BMC Complementary and Alternative Medicine*.

[7] Litscher G. (2006). Bioengineering assessment of acupuncture, part 2: monitoring of microcirculation. *Critical Reviews in Biomedical Engineering*.

[8] Litscher G. (2009). Ten years evidence-based high-tech acupuncture—a short review of peripherally measured effects. *Evidence-Based Complementary and Alternative Medicine*.

[9] Uchida S., Hotta H. (2008). Acupuncture affects regional blood flow in various organs. *Evidence-Based Complementary and Alternative Medicine*.

[10] Berardesca E., Elsner P., Maibach H. (1994). *Bioengineering of the Skin: Cutaneous Blood Flow and Erythema*.

[11] Larsson R., Öberg P. Å., Larsson S.-E. (1999). Changes of trapezius muscle blood flow and electromyography in chronic neck pain due to trapezius myalgia. *Pain*.

[12] Strøm V., Knardahl S., Stanghelle J. K., Røe C. (2009). Pain induced by a single simulated office-work session: time course and association with muscle blood flux and muscle activity. *European Journal of Pain*.

[13] Min S., Lee H., Kim S.-Y. (2015). Local changes in microcirculation and the analgesic effects of acupuncture: a laser doppler perfusion imaging study. *Journal of Alternative and Complementary Medicine*.

[14] Huang T., Wang R.-H., Zhang W.-B. (2012). The influence of different methods of acupuncture on skin surface perfusion. *Journal of Traditional Chinese Medicine*.

[15] Zhang W.-B., Wang L.-L., Huang T. (2008). Laser Doppler perfusion imaging for assessment of skin blood perfusion after acupuncture. *Medical Acupuncture*.

[16] Tsuchiya M., Sato E. F., Inoue M., Asada A. (2007). Acupuncture enhances generation of nitric oxide and increases local circulation. *Anesthesia and Analgesia*.

[17] Sandberg M., Larsson B., Lindberg L.-G., Gerdle B. (2005). Different patterns of blood flow response in the trapezius muscle following needle stimulation (acupuncture) between healthy subjects and patients with fibromyalgia and work-related trapezius myalgia. *European Journal of Pain*.

[18] Sandberg M., Lindberg L.-G., Gerdle B. (2004). Peripheral effects of needle stimulation (acupuncture) on skin and muscle blood flow in fibromyalgia. *European Journal of Pain*.

[19] Sandberg M., Lundeberg T., Lindberg L.-G., Gerdle B. (2003). Effects of acupuncture on skin and muscle blood flow in healthy subjects. *European Journal of Applied Physiology*.

[20] Litscher G., Wang L., Huber E., Nilsson G. (2002). Changed skin blood perfusion in the fingertip following acupuncture needle introduction as evaluated by laser Doppler perfusion imaging. *Lasers in Medical Science*.

[21] Higgins J. P. T., Green S. (2008). *Cochrane Handbook for Systematic Reviews of Interventions*.

[22] Wollina U., Abdel-Naser M. B., Mani R. (2006). A review of the microcirculation in skin in patients with chronic venous insufficiency: the problem and the evidence available for therapeutic options. *International Journal of Lower Extremity Wounds*.

[23] Bengtsson A. (2002). The muscle in fibromyalgia. *Rheumatology*.

[24] Sandberg M. L., Sandberg M. K., Dahl J. (2007). Blood flow changes in the trapezius muscle and overlying skin following transcutaneous electrical nerve stimulation. *Physical Therapy*.

[25] Sluka K. A., Walsh D. (2003). Transcutaneous electrical nerve stimulation: basic science mechanisms and clinical effectiveness. *The Journal of Pain*.

[26] Zhang Y., Ge H.-Y., Yue S.-W., Kimura Y., Arendt-Nielsen L. (2009). Attenuated skin blood flow response to nociceptive stimulation of latent myofascial trigger points. *Archives of Physical Medicine and Rehabilitation*.

[27] WHO (2007). *WHO International Standard Terminologies on Traditional Medicine in the Western Pacific Region*.

[28] Kimura K., Masuda K., Wakayama I. (2006). Changes in skin blood flow and skin sympathetic nerve activity in response to manual acupuncture stimulation in humans. *American Journal of Chinese Medicine*.

[29] Kistler A., Mariauzouls C., Kuhr C. (1996). Acute sympathetic responses elicited by acupuncture are pain-related and non-specific. *Forschende Komplementarmedizin*.

[30] Kang O.-S., Chang D.-S., Lee M.-H., Lee H., Park H.-J., Chae Y. (2011). Autonomic and subjective responses to real and sham acupuncture stimulation. *Autonomic Neuroscience: Basic and Clinical*.

[31] Lund I., Lundeberg T. (2006). Are minimal, superficial or sham acupuncture procedures acceptable as inert placebo controls?. *Acupuncture in Medicine*.

[32] Langevin H. M., Wayne P. M., MacPherson H. (2011). Paradoxes in acupuncture research: strategies for moving forward. *Evidence-Based Complementary and Alternative Medicine*.

[33] Cummings M. (2009). Modellvorhaben akupunktur—a summary of the ART, ARC and GERAC trials. *Acupuncture in Medicine*.

