# Supplementary material for: Changes of Local Blood Flow in Response to Acupuncture Stimulation: A Systematic Review
Source: Evid Based Complement Alternat Med. 2016 Jun 14;2016:9874207. doi: 10.1155/2016/9874207 (PMC4923553; doi:10.1155/2016/9874207)
Supplement: Supplementary file 1 — Details regarding study design, participants' characteristics, measurement accuracy, and experimental environment are shown. [file 9874207.f1.docx]

**Supplementary data 1. Details of study design and experimental environment**

| **Author (year)** | **Study design** | | **Participants characteristics** | | | **Measurement accuracy** | | | | **Experimental environment** | | | |
| --- | --- | --- | --- | --- | --- | --- | --- | --- | --- | --- | --- | --- | --- |
|  | **Cross-over** | **Washout period (time)** | **Age**  **Mean (range)** | **Gender** | **Experience of acupuncture** | **Posture during measurement** | **Movement control** | **Rest before measurement** | **Restriction (period)** | **Temperature (°C)** | **Humidity (%)** | **light** | **Others** |
| Min et al. (2015) | Yes | Meridian4 days interval | 22.8 | M, F | NR | Supine position (but not so comfortable to fall asleep) | Subject’s right hand with a kapok-filled vacuum cushion | 10min | Alcohol, caffeine, medication (12 hr) | 22-24 | NR | Light-conditioned room | Quiet room |
| Huang et al. (2012) | Yes | NR | 27 | M, F | NR | NR | Stable position as possible | lie down, 10 min | NR | 26 | 40-60 | NR | NR |
| Zhang et al. (2008) | Yes | Different days | (23-53) | M, F | NR | NR | NR | NR | NR | 26–28 | NR | NR | NR |
| Tsuchiya et al. (2007) | Yes | 1-week interval | 28±5 | NR | No | NR | NR | NR | Food (10 hr), drink (6 hr) | NR | NR | NR | NR |
| Sandberg et al. (2005) | Yes | 2 days-2 weeks interval (at the same time) | 36/38/49^a^ | F | Yes^b^ | Seated position | Sit as relaxed as possible and not move around | NR | Food, coffee, chocolate, tea, exercise (2 hr), smoking^c^ | 23–25 | NR | Moderate light | Quiet room |
| Sandberg et al. (2004) | Yes | 2-5 days interval (at the same time) | 40 (24-54) | F | Yes^b^ | Supine position | NR | 30 min | Food, coffee, chocolate, tea, exercise (2 hr)  smoking^c^ | 23 ± 1 | NR | Moderate light | Quiet room |
| Sandberg et al. (2003) | Yes | 2-7 days interval (at the same time) | 38 | F | Yes^b^ | Supine position | NR | 30 min | Food, coffee, chocolate, tea, exercise (2 hr)  smoking^c^ | 23 ± 1 | NR | Moderate light | Quiet room |
| Litscher et al. (2002) | Yes | 2 hours-1 day interval | 25 (19-59) | M, F | NR | Supine position in a comfortable position | NR | 10 min | Medication^c^ | NR | NR | NR | NR |

^a^Healthy subjects/fibromyalgia patients/trapezius myalgia patients

^b^Those subjects who had no previous experience of acupuncture had an initial individual visit to the department to experience the needling

^c^Exclusion criteria

F, female; hr, hours; M, male; min, minutes; NR, not reported.
